# 

**Published:** 1907-05-04

**Authors:** 


### GENERAL PRACTITIONERS' CONTRIBUTIONS.

### Important.

We propose to devote a special page to General Practitioners' Contributions. We therefore invite from practitioners contributions based upon their experience in the management of cases, and in the treatment and diagnosis of disease; especially shall we be prepared to welcome articles dealing, practically, with treatment, and with the use and value of new remedies and methods.

No article should exceed 1,100 words in length, and, if accepted, one guinea will be paid to the writer after publication. Each communication should be accompanied by a stamped directed envelope for the return of the MS. if found unsuitable. See coupon on Special Supplement.

### The Relaxations of Medical Men.

We shall also be glad to pay for accepted contributions, from any member of the profession, on the subject of the relaxations of practitioners. This opens up a wide field, as it includes natural history, photography, sport, indoor recreations, and motoring. Whenever possible, original illustrations and photographs should be sent with the MS.

### Suggestions Invited.

The Editor will welcome suggestions for the establishment of any new section in The Hospital, and will be glad to supply information on any subject of interest or importance to members of the profession in any part of the world.

### NOTICES AND ANSWERS TO CORRESPONDENCE.

All MSS., letters, books for review, and other matters intended for the Editor, should be addressed to :—

The Editor,
The Hospital Building,
28 and 29 Southampton Street,
Strand, London, W.C.

### BUSINESS NOTICES.

Letters relating to the Publishing, Sale and Advertisement Departments must be addressed to the Manager (*not to the Editor*) :—

The Manager,
The Hospital Building,
28 and 29 Southampton Street,
Strand, London, W.C.

### Annual Subscriptions.

The rate of annual subscriptions to The Hospital, either direct from the Office or through agents, is :—

| | |
|---|---|
| For the United Kingdom ... ... ... | 13s. a year |
| To the Colonies and Abroad ... ... | 17s. a year |

inclusive of postage in each case.

### Subscriptions.

Subscriptions are payable in advance. Cheques and Postal Orders should be made payable to :—

The Scientific Press, Ltd., and should be crossed : London and County Banking Co., Ltd.

THE HOSPITAL *May* 4, 1907.

*Name* ..........................................................

*Address* ..........................................................

..........................................................

This Coupon must accompany manuscript or contributions intended for The Hospital.

